# Comparative analysis of diagnostic expectations between adults referred for ADHD and autism assessment: a thematic analysis

**DOI:** 10.3389/fpsyt.2026.1780570

**Published:** 2026-06-11

**Authors:** Marios Adamou, Lydia Wharton

**Affiliations:** 1 School of Human and Health Sciences, University of Huddersfield, Huddersfield, United Kingdom; 2 South West Yorkshire Partnership NHS Foundation Trust, Wakefield, United Kingdom

**Keywords:** ADHD, autism, diagnostic expectations, qualitative research, service design, thematic analysis

## Abstract

**Background:**

Recent trends in neurodevelopmental services promote joint assessment pathways combining ADHD and Autism services. However, no study has systematically compared diagnostic expectations between adults seeking ADHD versus Autism assessment.

**Methods:**

This comparative qualitative study analysed expectations from 100 adults referred for ADHD assessment, comparing findings with published Autism data (n=60) from the same Trust. Both samples were of adults referred for assessment rather than diagnostically confirmed cohorts. Two open-ended questions during face-to-face appointments: “What do you hope to gain from the assessment?” and “How do you think receiving a diagnosis would help you?” ADHD responses underwent reflexive thematic analysis using Braun and Clarke’s six-phase framework, with comparison against published Autism themes.

**Results:**

Analysis revealed fundamentally divergent expectation patterns organised around contrasting meta-themes: “Seeking Legitimisation and Practical Solutions” (ADHD) versus “Understanding Self and Explaining Difference” (Autism). Five conceptual dichotomies distinguished pathways: (1) pharmacological versus psychosocial intervention paradigms (47% ADHD mentioned medication versus 1.7% Autism); (2) diagnostic certainty versus depth of understanding (31% ADHD versus 75% Autism seeking understanding); (3) passive hope for understanding versus active educator positioning (3% ADHD versus 63% Autism expecting to explain to others); (4) deficit versus difference self-concepts (15% ADHD used harsh self-descriptors versus 1.7% Autism); (5) internal overwhelm versus social-environmental mismatch phenomenology (10% ADHD described “racing mind” versus 0% Autism). These differences persisted across demographic subgroups.

**Conclusions:**

Adults seeking ADHD versus Autism assessment hold fundamentally different expectations requiring differentiated clinical responses. Findings provide empirical evidence for separate rather than combined diagnostic pathways, with condition-specific pre-assessment counselling, post-diagnostic support, and clinical expertise development. Generic ‘neurodevelopmental’ provision risks meeting no one’s actual needs.

## Introduction

Autism Spectrum Disorder (ASD) and Attention-Deficit/Hyperactivity Disorder (ADHD) are lifelong neurodevelopmental conditions with distinct and occasionally overlapping characteristics (1). ASD includes difficulties in social communication and interaction, along with restricted and repetitive behaviours, and ADHD is defined by ongoing patterns of inattention, hyperactivity, and impulsivity that impair daily functioning (2). Although diagnoses commonly occur in childhood, many adults go unrecognised for years. Over time, social, professional, or emotional demands, especially in unstructured or high-stress situations, can lead to challenges that prompt the search for diagnostic confirmation (3, 4).

Recent years have seen a substantial rise in adults pursuing diagnoses for ASD and ADHD. In England, as of June 2025, 236,225 people awaited ASD assessments, with approximately 10,000 new referrals each month (5–7). For ADHD, 668,370 people remained on waiting lists as of October 2025 (8). This expansion in referrals indicates increased public awareness of these conditions in adulthood and societal recognition of neurodevelopmental diversity across life stages. Extended waiting times for assessments aggravate mental health concerns, including anxiety, depression, and social isolation, whilst restricting access to essential support services that could improve quality of life (9, 10).

Obtaining an ASD or ADHD diagnosis in adulthood represents a significant experience for many. It often provides relief by contextualising persistent difficulties such as social interaction, emotional regulation, executive functioning, organisation, or time management as neurodevelopmental traits rather than personal deficiencies. This perspective promotes self-acceptance (11) and facilitates access to tailored interventions, including accommodations, psychological support, psychoeducation, medication, and workplace adjustments (12, 13). The diagnostic process extends beyond therefore a clinical process to a personal milestone that reshapes identity, self-worth, and life direction.

Research has explored diagnostic experiences in Autism, identifying contributing elements (14–19). Phenomenological studies found themes such as feeling different, emotional shifts, self-acceptance efforts, strategy development, and future contemplation (20). An important expectation is clearer self-understanding, with reviews emphasising desires for improved identity and relationship awareness prior to diagnosis (18, 21). The emotional trajectory of diagnosis is important as this understanding may improve well-being and self-acceptance, with later-diagnosed women often reporting relief through explanatory frameworks (22). Yet, responses can also include grief or loss alongside clarity, with challenges in identity adjustment and stigma management. Investigations highlight persistent feelings of difference and regrets over delayed recognition, noting that early diagnosis aids acceptance whilst later timing hinders identity formation (23, 24). Certain adults anticipate benefits beyond personal gains, viewing diagnosis as a tool to enhance social acceptance amid stigma that fosters shame or stereotyping, potentially altering societal and professional perceptions (25).

Qualitative research on ADHD highlights desires for cognitive stability, functional improvement, relief from self-blame, and access to medication (26, 27). Studies indicate that ADHD adults often seek diagnosis during periods of crisis or functional breakdown, with medication expectations prominent (28). Research emphasises the quest for legitimisation against moral attributions of laziness or incompetence, suggesting ADHD stigma manifests differently from Autism stigma (29–32). For ADHD, additional drivers include access to stimulant medication and recognition of hereditary patterns, particularly after children’s diagnoses (33). However, ADHD diagnostic expectations remain less extensively researched than Autism expectations, with fewer studies examining the adult diagnosis-seeking experience. The empirical literature on the pre-assessment diagnostic expectations of adults presenting with concurrent ADHD and Autism concerns is, to our knowledge, undeveloped, and the present study does not address that population directly. This gap is itself one of the reasons that a clear, condition-specific evidence base for adults entering ADHD and Autism referral pathways separately is required as a first step before a comparative dual-presentation study can be meaningfully designed.

Motivations for seeking a diagnosis vary and are influenced by personal and external factors. People may address misunderstandings in relationships, employment, or education; secure accommodations or assistance; or gain insight into past experiences. Cultural and societal influences shape perceptions of these diagnoses and their community implications (34). ADHD remains primarily positioned within the medical model presumably as it is considered as treatable with cultural narratives emphasising pharmacological treatment for biological deficits. The neurodiversity movement, whilst increasingly influential for Autism, has developed less extensively for ADHD, leaving people without well-established alternative frameworks to challenge deficit narratives (35). This divergence in cultural positioning may predict different forms of stigma internalisation. ADHD is perceived more as moral failure whilst Autism as social oddity requiring identity affirmation. Common across both conditions is the hope that a formal diagnosis may reduce stigma and improve social or professional acceptance (11, 36, 37).

However, no study has directly and systematically compared the expectations of adults seeking ADHD versus ASD diagnoses using parallel qualitative methodology. This evidence gap has direct clinical implications. Many healthcare services consolidate ADHD and ASD assessments into integrated neurodevelopmental pathways to improve efficiency (38, 39). Recent healthcare policy has increasingly promoted such integrated approaches, assuming that people referred for either condition share similar needs and expectations, potentially improving efficiency through unified protocols. However, if expectations differ fundamentally, integrated pathways risk providing generic, condition-ambiguous support that fails to meet specific needs, such as medication counselling for ADHD or identity-focused support for ASD. Without empirical evidence comparing expectations across conditions, service design decisions rest on assumptions about shared needs rather than data about actual patient priorities. The question of whether separate or integrated assessment pathways better serve patient needs remains unanswered.

This study adopts a critical realist epistemological stance (40), which holds that participant accounts represent meaningful subjective experiences (the empirical level) that arise from real but mediated underlying mechanisms (the actual and real levels), including cultural narratives, healthcare structures, and neurodiversity discourse. In contrast to a purely constructivist position, which would treat participant accounts as wholly discursive constructions to be analysed for their performative functions, the critical realist position taken here treats participant accounts as informative about their subjective experience whilst explicitly acknowledging that those experiences are shaped by structural and cultural mechanisms that are themselves real. In practice, this stance guided coding by permitting both semantic codes (what participants said) and latent codes (the cultural and structural assumptions implicit in what they said), and by allowing interpretation to move between the level of individual meaning-making and the level of the broader medical and identity-model frameworks within which participants are situated.

The aims of this study include conducting a comparative thematic analysis of the expectations of adults referred for ADHD and ASD assessments within the same healthcare trust. The study employs identically collected qualitative information and a single analyst to ensure methodological parity. The study addresses two research questions:

1. What are the primary expectations of adults seeking ADHD assessment, and how do they compare to those seeking ASD assessment?
2. How do these expectations reflect broader condition-specific narratives, stigma experiences, and intervention paradigms?

Findings will inform whether separate or integrated assessment pathways better meet patient needs and contribute to a deeper theoretical understanding of neurodevelopmental diagnosis in adulthood. By providing the first systematic comparison of ADHD diagnostic expectations with published Autism data using the same questions from the same Trust, this study addresses a critical evidence gap with immediate implications for service design and clinical practice.

## Methods

### Study design and setting

This comparative qualitative study used reflexive thematic analysis (41) to examine diagnostic expectations among adults referred for neurodevelopmental assessment. Data were drawn from two cohorts: 100 adults referred for ADHD screening and triage assessment, and 60 adults referred for Autism diagnostic assessment, both within the South West Yorkshire Partnership NHS Foundation Trust. It is important to clarify that the Autism cohort data derive from a previously published study (Adamou et al., 2025) (21), and that the present paper compares the newly analysed ADHD findings against those published Autism themes using the identical first question (“What do you hope to gain from the assessment?”). The comparative claims in this paper therefore concern participant-generated expectations articulated at the pre-assessment stage and not claims about the availability or effectiveness of treatments for either condition.

Data collection occurred within routine clinical pathways. The ADHD cohort attended 45-minute face-to-face assessment appointments between December 2024 and February 2025. The Autism cohort attended 30-minute face-to-face assessment appointments between January and June 2023, as previously described (21). Both services serve a mixed urban and rural population of approximately 2 million adults. The study was approved by the Trust’s Service Evaluation and Quality Improvement Committee.

### Participants and sampling

#### ADHD sample

Consecutive referrals for adult ADHD assessment between December 2024 and February 2025 were screened for inclusion. Eligibility required adults aged 18 years and over referred for specialist ADHD opinion, with no previous formal ADHD diagnosis. Comorbidities or other diagnoses were not exclusionary. The first 100 eligible people formed the sample. The sample was selected to have an equal number of men and women and were 50 men and 50 women.

Data saturation was assessed across the full dataset of 100 participants. While distinct semantic nuances emerged as late as Participant 65 (who contributed specific medication requests), the analysis continued through to the final participant. The coding of the final cohort, exemplified by Participant 99 (Validation) and Participant 100 (Gatekeeping) confirmed that these later accounts reinforced established themes rather than generating new codes, indicating that robust theoretical saturation was achieved within the complete sample. The sample of 100 provided adequate data for demographic stratification whilst ensuring comprehensive thematic coverage.

#### Autism sample

The methodology for the Autism sample replicates that of the published study (21). Adults aged 18 years and over referred for Autism assessment between January and June 2023 were eligible if they had no previous Autism diagnosis or global learning disability. Through systematic recruitment, 60 participants (30 men, 30 women) were included. Data saturation was confirmed during the original analysis when no new themes emerged from subsequent participant responses.

#### Participant characteristics

The study included 160 participants across two diagnostic pathways and included 100 adults referred for ADHD assessment and 60 adults referred for Autism assessment. Both groups were of people referred for an assessment that of people with an established diagnosis. The participants were asked about their expectations at the pre-assessment screening and triage stage, and at the point of data collection none held a confirmed ADHD or Autism diagnosis. The classifying variable between the two groups is therefore the referral pathway, which is the clinically and administratively relevant distinction for the service design questions addressed by this study.

Both samples were selected to have identical gender distribution (50% male, 50% female). The ADHD sample had a mean age of 34.7 years (SD = 10.5, range 18-63), whilst the Autism sample had a mean age of 32.9 years (SD = 12.1, range 18-68). Age distribution differed between groups, with the Autism sample skewing younger (53.3% aged 18-30) compared to the ADHD sample (37.0% aged 18-30, χ²=4.12, p=0.042). The majority of both samples fell within working age (18–50 years): 93% of the ADHD sample and 88.3% of the Autism sample. Detailed demographic characteristics are presented in **Table 1**.

**Table 1:** Demographic characteristics of ADHD and autism samples.

Characteristic	ADHD (n=100)	Autism (n=60)	P-value
Gender, n (%)			1.000^a^
Male	50 (50.0)	30 (50.0)	
Female	50 (50.0)	30 (50.0)	
Age, years			
Mean (SD)	34.7 (10.5)	32.9 (12.1)	0.298^b^
Median	33	30	
Range	18-63	18-68	
Age group, n (%)			0.042^a^
18–30 years	37 (37.0)	32 (53.3)	
31–50 years	56 (56.0)	21 (35.0)	
51+ years	7 (7.0)	7 (11.7)	
Ethnicity, n (%)			0.055^a^
White British	91 (91.0)	48 (80.0)	
Minority Ethnic	9 (7.0)	12 (20.0)	

^a^Fisher’s exact test. ^b^Independent samples t-test.

The age asymmetry between the two samples (Autism: 53.3% aged 18–30; ADHD: 37.0% aged 18–30; p=0.042) may be an important differentiator because younger adults may be expected to articulate identity-formation themes more readily than older adults. We addressed this in two ways. First, we conducted age-stratified analyses (**Table 2**) and found that the main between-group differences (medication, self-understanding, explaining to others, racing mind) persist within each age stratum, with Autism participants in every age band reporting significantly higher self-understanding than ADHD participants in the same age band (72%, 76%, and 86% versus 30%, 23%, and 29% respectively). Second, the magnitude of the main differences is such that an age-driven contribution alone cannot plausibly account for them. We therefore retain the interpretation that the divergence reflects pathway-related rather than age-related factors, whilst acknowledging that the Autism sample’s younger skew may have contributed to the salience of identity themes at the margin.

**Table 2:** Age-stratified expectations by diagnostic group.

Expectation	ADHD 18-30 (n=37)	ADHD 31-50 (n=56)	ADHD 51+ (n=7)	Autism 18-30 (n=32)	Autism 31-50 (n=21)	Autism 51+ (n=7)
Medication	43	49	57	3	0	0
Self-understanding	30	23	29	72	76	86
Explaining to others	3	4	0	63	67	57
Educational support	35	14	0	44	19	14
Workplace concerns	38	50	29	38	48	14
Parenting concerns	3	25	0	3	14	14
Racing mind	5	9	71	0	0	0

### Data collection

#### Procedure

For both ADHD and Autism samples, data collection followed the same procedure during face-to-face appointments (45 minutes for ADHD, 30 minutes for Autism). Participants were verbally asked two open-ended questions about their expectations for the assessment: (1) “What do you hope to gain from the assessment?” and (2) “How do you think receiving a diagnosis would help you?” Their verbal responses were recorded by clinicians on paper as part of routine clinical documentation. No audio or video recordings were made, and participants did not provide written responses directly. This formed part of routine clinical practice to understand patient expectations.

We are also aware that patients reported their responses verbatim to their clinicians, who wrote them down, rather than recorded them on a device such as audio, so the results could have been subject to selection, compression or summary in ways that may be different from how the patient spoke, even after training on appropriate procedure and standard practice. Two features of the design partially mitigate this. First, the same procedure was applied across both cohorts, so any systematic clinician filter would be expected to operate symmetrically rather than to generate the asymmetries we report. Second, the data preserve sufficient verbatim phrasing (for example, the speed metaphors in the racing-mind theme and the morally laden self-descriptions in the rejecting-negative-self-attributions theme) to support the principal interpretive claims. Nonetheless, audio-recorded and transcribed interviews would yield richer data and remain the methodological gold standard for future work in this area.

#### Ethical consent

For both cohorts, verbal informed consent for the anonymised use of clinical data for service evaluation was obtained during the appointment as part of standard care. This retrospective analysis used only fully anonymised data.

#### Data preparation for comparative analysis

Responses to both questions were extracted, anonymised, and compiled into datasets. A unique identifier, age, and gender were recorded for each participant. All identifying details were removed. For this comparative analysis, findings from both questions were integrated to provide a comprehensive understanding of participant expectations.

#### Methodological limitations

We acknowledge some methodological limitations. First, the different data collection periods (Autism January-June 2023, ADHD December 2024-February 2025) mean samples were collected 12–18 months apart. Whilst no major service changes occurred during this period, temporal effects cannot be entirely excluded.

We further acknowledge that public and media discourse on adult ADHD and Autism evolved during the period between the two collection periods, and we cannot exclude the possibility that some component of the divergence we report reflects a shift in awareness or framing in the wider environment rather than a stable difference between the two referral pathways. We note, however, that the cultural and structural factors driving such awareness shifts (for example, the differential development of neurodiversity framing for Autism and the medical-model positioning of ADHD) are themselves part of the explanatory account we offer and would not be expected to invert the direction of the main findings.

Second, brief responses from routine clinical practice may not capture full complexity of expectations. However, this naturalistic approach provides high ecological validity as these are actual patient statements from real clinical encounters.

### Analytical approach

We employed reflexive thematic analysis following Braun and Clarke’s six-phase framework (41). This approach was selected for its flexibility, theoretical accessibility, and suitability for comparative analysis. The analysis followed an inductive, data-driven approach rather than applying pre-existing theoretical frameworks. We employed both semantic coding (capturing explicit meaning in participant responses) and latent coding (identifying underlying concepts, assumptions, and implications). The analysis was informed by critical realist epistemology (40), acknowledging that participant responses represent meaningful expressions of expectations whilst recognising that these expectations are shaped by cultural, social, and structural contexts.

We note that only the ADHD sample (n=100) was analysed by the lead analyst (MA) for this study. The Autism data (n=60) were taken directly from the previously published analysis (Adamou et al., 2025), which used the same Question 1 (“What do you hope to gain from the assessment?”) from the same Trust. This approach enabled valid comparison between ADHD expectations and published Autism expectations whilst avoiding re-analysis of the Autism dataset.

#### Phase 1: familiarisation

The analyst read all 160 responses multiple times to achieve deep familiarity with both datasets. Initial observations were documented in analytical memos noting apparent patterns, variations, and potential areas for comparison.

#### Phase 2: systematic coding

All ADHD responses (n=100) were coded line-by-line using both semantic and latent approaches. Each meaningful unit of text received one or more codes capturing its content and underlying meaning. Coding was conducted using NVivo qualitative data analysis software (QSR International Pty Ltd. Version 14). The analyst maintained a codebook documenting all codes generated, their definitions, and exemplar quotes. For the Autism sample, coding had been completed as part of the published study (Adamou et al., 2025); the present study used the published themes and prevalence data for comparison purposes.

#### Phase 3: initial theme generation

ADHD codes were reviewed systematically to identify patterns and potential themes. Related codes were clustered based on conceptual similarity, with attention to both prevalence (how many participants mentioned the concept) and centrality (how integral the concept appeared to participant expectations). Provisional themes were generated for the ADHD sample, then compared systematically with the published Autism themes (21) to identify commonalities and differences.

#### Phase 4: Theme review and refinement

Provisional ADHD themes were reviewed against the ADHD dataset (n=100) to ensure they adequately captured the range and pattern of participant responses. Themes were refined, merged, or split based on this review. Particular attention was paid to confirming or disconfirming ADHD-specific themes through systematic examination of whether similar patterns appeared in the published Autism data.

#### Phase 5: theme definition and naming

Each ADHD theme was clearly defined and named. Detailed descriptions articulated what each theme represented and how it contributed to understanding ADHD participants’ expectations. Theme names were developed to capture essential meaning whilst remaining accessible. For ADHD, themes were organised hierarchically, identifying meta-themes capturing overarching patterns. For the Autism sample, we used the published themes and definitions from our previous publication (21): Personal Growth, Explanation for Others, Access to Support, Inclusivity and Acceptance, and Progress in Life Domains.

#### Phase 6: producing the analysis

The final ADHD analysis integrated quantitative description (theme prevalence, frequencies) with qualitative interpretation (meaning, significance, implications). For each ADHD theme, we calculated the proportion of participants whose responses contained relevant codes. These prevalence rates were then statistically compared with the published Autism theme prevalence rates (21) using chi-square or Fisher’s exact tests. Exemplar quotes from ADHD participants were selected based on typicality, clarity, and diversity of expression within themes. Comparative analysis examined five dimensions: theme prevalence, language use, social positioning, temporal orientation, and intervention expectations.

### Comparative and demographic analysis

Following thematic analysis, we conducted stratified analysis examining whether theme prevalence varied by age group (18-30, 31-50, 51+ years) or gender (male, female) within each diagnostic group. Statistical analyses were performed using SPSS Statistics (IBM Corp. Version 29.0). Chi-square tests were used for comparisons where expected cell counts were ≥5 in all cells; Fisher’s exact test was used when any expected cell count was <5. Statistical significance was set at p<0.05 (two-tailed). For the eight major theme comparisons between diagnostic groups, we applied Bonferroni correction for multiple comparisons (corrected α = 0.006). Both corrected and uncorrected p-values are reported for transparency.

Age groups were defined based on developmental and life stage considerations: 18–30 years (young adults in education and early career), 31–50 years (established careers and active parenting), 51+ years (later career and older adulthood). These categories showed adequate sample sizes for statistical comparison in both samples.

### Researcher positionality and reflexivity

The lead analyst (MA) is a male consultant psychiatrist and Professor of Psychiatry with clinical expertise in adult ADHD and Autism assessment, employed by the Trust providing services to both samples. This insider position provided familiarity with assessment processes, diagnostic criteria, and clinical presentations, enabling close interpretation of participant responses, but it also carried a specific risk of medical-model drift, that is, a tendency to read participant accounts through the lens of disorder, deficit, and pharmacological intervention rather than through the lens of difference, identity, and accommodation. Three steps were taken to mitigate this. First, the analyst maintained reflexive memos throughout coding in which clinical assumptions were named and questioned, with explicit attention to whether a code was being generated by the data or imposed from a clinical framework. Second, the analyst’s previous published work was used as a deliberate counterweight to medical-model framing, and the Autism comparative themes (drawn from the published study) functioned as a second perspective external to the present coding process. Third, the co-author reviewed the emerging thematic structure independently and challenged interpretations that appeared to reflect clinical default rather than participant voice. We acknowledge that complete neutralisation of analyst positionality is not achievable, and that a non-clinician analyst, or an analyst from an autistic or ADHD self-advocacy background, would likely have generated a partially different reading of the same data; the findings should be read with that in mind.

### Ethical considerations

For both samples, this was a retrospective secondary analysis of anonymised data collected as part of routine clinical practice. In accordance with the Declaration of Helsinki, the Institutional Review Board granted a waiver for formal ethics approval. Verbal informed consent for service evaluation was obtained during routine clinical appointments.

All data were fully anonymised before the analysis. All quotes were reviewed to ensure they contained no identifying information.

### Patient and public involvement

This analysis emerged from direct statements of different expectation patterns. No patient or public involvement occurred in study design or initial analysis. We plan to disseminate findings to patient groups and advocacy organisations, seeking feedback on interpretations.

### Reporting standards

This manuscript follows COREQ guidelines (42) and SRQR standards (43). The full anonymised dataset is available on reasonable request to the corresponding author.

## Results

### Overview of comparative analytic approach

This study employed reflexive thematic analysis to examine diagnostic assessment expectations among 100 adults referred for ADHD assessment, with comparative analysis against previously published data from 60 adults referred for Autism assessment (21). Both samples were of adults at the pre-assessment screening and triage stage rather than diagnostically confirmed cohorts; the comparative findings described below therefore concern the expectations of individuals as they enter the ADHD or Autism referral pathway. Both samples were asked two open-ended questions about their expectations: “What do you hope to gain from the assessment?” and “How do you think receiving a diagnosis would help you?” Analysis revealed fundamentally divergent expectation patterns organised around contrasting meta-themes: “Seeking Legitimisation and Practical Solutions” for ADHD versus “Understanding Self and Explaining Difference” for Autism. These meta-themes represent distinct orientations toward diagnosis, one seeking external validation and concrete intervention, the other pursuing identity development and social understanding.

### Autism comparative framework

The Autism sample analysis, previously published (21) identified five major themes under the meta-theme ‘Understanding Self and Explaining Difference’: (1) Personal Growth (including insight and awareness 75%, identity formation 67%, sense of agency 58%, closure 50%); (2) Explanation for Others (seeking understanding from others 63%, educating others about Autism 25%); (3) Access to Support (reasonable adjustments 53%, mental health support 45%); (4) Inclusivity and Acceptance (validation 70%, normalisation 55%, self-acceptance 50%); (5) Progress in Life Domains (interpersonal relationships 33%, employment/education progression 42%). These themes provided the comparative framework against which ADHD expectations were analysed.

### ADHD-specific themes: seeking legitimisation and practical solutions

**Figure 1:**
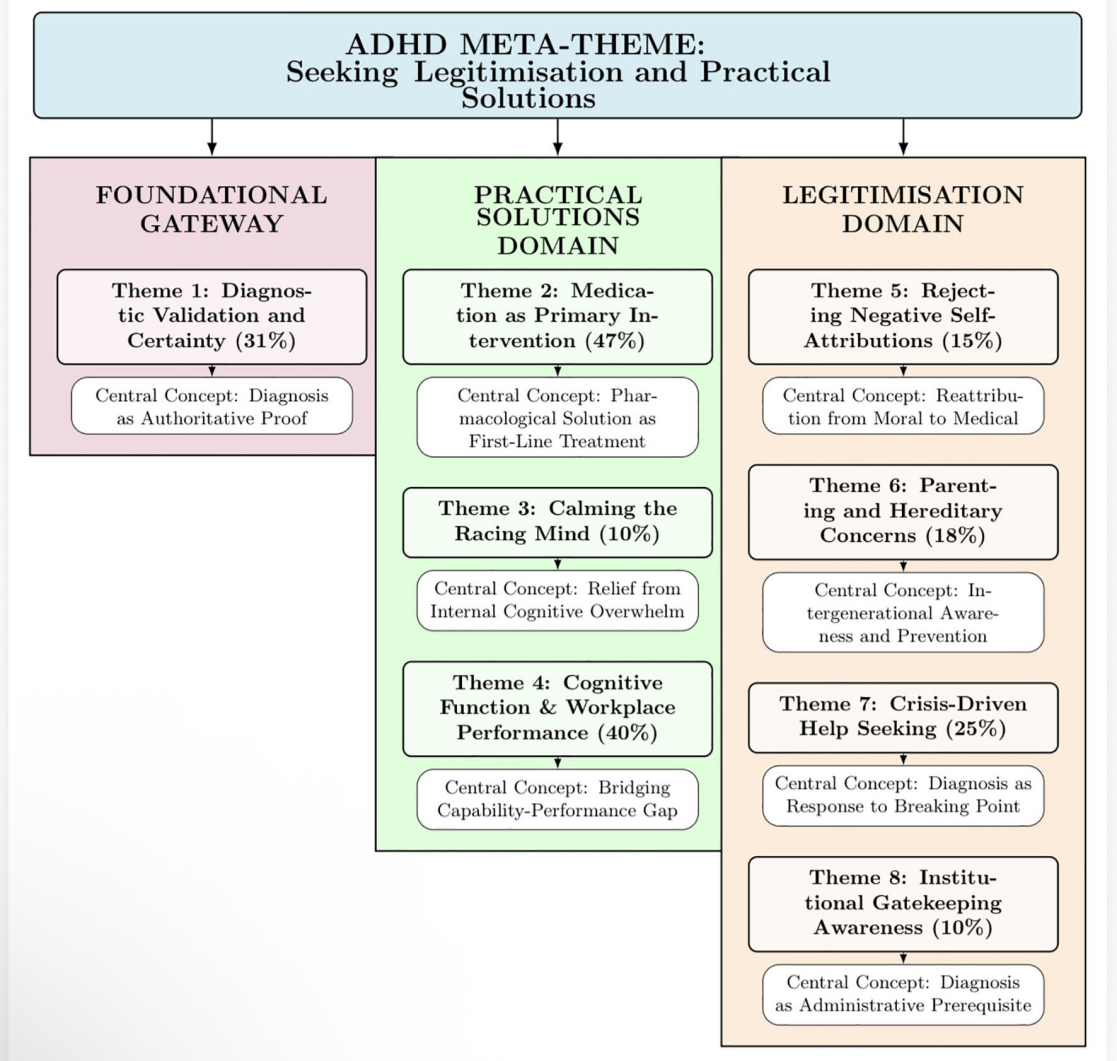
Hierarchical Thematic Structure of ADHD Diagnostic Expectations.

Analysis of the ADHD sample revealed eight central themes organised hierarchically under the overarching meta-theme of legitimisation and practical solutions (**Figure 1**). The thematic structure demonstrates how diagnostic certainty serves as the foundational gateway enabling both psychological validation and access to practical interventions, particularly pharmacological treatment.

#### Theme 1: diagnostic validation and certainty (31%)

Nearly one-third of ADHD participants (31%) explicitly sought definitive diagnostic confirmation, expressing a need for categorical certainty that far exceeded Autism participants’ emphasis (10%, p=0.002). Participants employed binary, proof-oriented language: “a yes or no answer,” “to know for sure,” or most tellingly, “proof.” This conceptualisation of diagnosis as evidentiary validation contrasted sharply with Autism participants’ framing of diagnosis as self-understanding.

The certainty sought served dual psychological and practical functions. Psychologically, it represented external authority to counteract internal doubt: “Would like to know if he had ADHD or not. To look for support and help/assistance” (P55, male, 34 years). Practically, it functioned as necessary documentation for institutional access: “A yes or no so work know” (P34, female, 35 years).

#### Theme 2: medication as primary intervention (47%)

Medication expectations dominated the ADHD sample, with 47% spontaneously mentioning pharmacological treatment as a primary hope 28 times more frequently than Autism participants (1.7%, p<0.001). Participants demonstrated sophisticated medication awareness, often differentiating ADHD-specific treatments from previous unsuccessful interventions: “Medication and be on the right medication, ideally wean off antidepressants” (P19, male, 31 years).

Three distinct medication narratives emerged: (1) Treatment optimisation (“the right medication”); (2) Switching from ineffective treatments, particularly antidepressants mentioned by 9% of participants; and (3) Specific pharmacological knowledge: “Wants Ritalin” (P65, male, 49 years).

#### Theme 3: calming the racing mind (10%)

A distinctive phenomenological experience emerged exclusively in ADHD responses, with 10% of participants using vivid speed metaphors to describe relentless, exhausting cognitive activity. This theme was completely absent from Autism responses (p=0.008). Participants described minds operating at unsustainable velocities: “She feels 1000mph all the time” (P31, female, 30 years), “Wants medication to stop the overwhelm and being 100mph” (P67, female, 29 years).

The desired outcome was fundamental state change: slowing, quieting, achieving mental stillness. Participants sought “peace and quiet for 10 minutes would be amazing” (P79, female, 49 years).

#### Theme 4: Cognitive function & workplace performance (40%)

Two-fifths of ADHD participants (40%) focused specifically on improving discrete cognitive functions particularly concentration, focus, and task completion with workplace performance as the primary context. Participants articulated a frustrating discrepancy between perceived capability and actual output: “Wants to be able to focus at work because is a smart person, wants to have single thoughts” (P12, female, 36 years).

This functional, performance-based orientation contrasted fundamentally with Autism participants’ emphasis on environmental accommodations.

#### Theme 5: rejecting negative self-attributions (15%)

Fifteen percent of ADHD participants used strongly negative, morally laden self-descriptors that they hoped diagnosis would medically refute. This pattern was seven times more frequent than in Autism participants (1.7%, p=0.006). Participants employed terms reflecting character judgment: “stupid,” “lazy,” “insane,” “crazy,” and most starkly, “shit as a person” (P94, female, 31 years).

Diagnosis functioned as exonerating evidence: “Something in my head I would know it’s not just me being stupid” (P3, male, 31 years).

#### Theme 6: parenting and hereditary concerns (18%)

Nearly one-fifth of ADHD participants (18%) spontaneously mentioned children, expressing concern about hereditary transmission and desire to support potentially affected offspring. This theme peaked in middle-aged participants (31–50 years: 25%) and was more than twice as prevalent as in Autism participants (8%, p=0.077). Participants framed parenting through a lens of shared neurobiology: “Knows a hereditary element, I see some things in my boy, if I have it I can help him” (P3, male, 31 years).

#### Theme 7: crisis-driven help seeking (25%)

A quarter of ADHD participants described acute crises precipitating assessment, using language of urgency, deterioration, and imminent collapse. ADHD participants reported functional systems nearing failure: “Expecting twins and now feels life is about to get much harder and needs help” (P61, male, 35 years), “Cannot cope anymore” (P50, male, 54 years), “Getting sacked over nothing” (P84, female, 38 years).

Temporal markers indicated present-moment urgency: “getting worse,” “life about to get much harder.”

#### Theme 8: institutional gatekeeping awareness (10%)

Ten percent of ADHD participants explicitly recognised institutional systems requiring formal diagnosis before support access. This was twice as prevalent as in Autism participants (5%, p=0.317). Participants articulated clear systemic barriers: “Her work have told her they won’t give her support unless she has a diagnosis” (P28, female, 31 years).

### Comparative analysis: fundamental dichotomies in expectation

Analysis revealed five core dichotomies distinguishing ADHD from Autism diagnostic expectations (**Table 3**):

**Table 3:** Core dichotomies distinguishing ADHD from Autism diagnostic expectations.

Theme/expectation	ADHD (n=100)	Autism (n=60)	Difference %	P-value
Medication	47	1.7	+45.3	<0.001*
Self-understanding	26	75	-49.0	<0.001*
Explaining to others	3	63	-60.0	<0.001*
Diagnostic certainty	31	10	+21.0	0.002*
Negative self-attributions	15	1.7	+13.3	0.006*
Racing mind	10	0	+10.0	0.008
Workplace accommodations	40	53	-13.0	0.104
Parenting concerns	18	8	+10.0	0.077
Crisis language	25	--	--	--
Institutional gatekeeping	10	5	+5.0	0.317

*p<0.006 (Bonferroni-corrected α for 8 primary comparisons).

1. Legitimisation vs. Identity Formation: ADHD participants pursued external legitimisation, proof to counteract accusations of laziness or stupidity. Autism participants sought identity affirmation, a positive framework for self-understanding.
2. Intervention Paradigm: Pharmacological vs. Psychosocial: ADHD participants overwhelmingly (47%) framed pharmacological intervention as primary hope. Autism participants focused on psychosocial and systemic solutions such as understanding, accommodations, educating others.
3. Temporal Orientation: Present Crisis vs. Lifelong Narrative: ADHD expectations were precipitated by acute, present-moment crises. Autism expectations were part of reflective, lifelong processes of making sense of cumulative experiences.
4. Self-Concept: Deficit vs. Difference: ADHD participants described themselves in terms of deficit and moral failing. Autism participants used language of neurological and social difference.
5. Social Goal: Normalisation vs. Explanation: ADHD participants sought normalisation, to “feel normal” and “do normal things.” Autism participants sought explanation and education to help others understand their different operating system.

### Demographic variations in expectation patterns

#### Age-stratified expectations

Age significantly influenced expectation patterns in both groups (**Table 2**), though baseline differences persisted within each age stratum. Younger adults (18–30 years) in both groups emphasised educational support, though ADHD participants showed stronger medication expectations (43% vs. 3%). Middle-aged adults (31–50 years) focused on workplace performance, with ADHD participants showing particularly high concern (50% vs. 38%) and continued strong medication expectations (49% vs. 0%). Parenting concerns peaked in ADHD middle adulthood (25% vs. 5%). Older adults (51+ years) in both groups prioritised retrospective understanding, though ADHD participants maintained medication expectations (57% vs. 0%).

#### Gender-stratified expectations

Gender differences were more pronounced in the ADHD sample than the Autism sample (**Table 4**). ADHD males emphasised diagnostic certainty (44% vs. 28%) and cognitive function (44% vs. 28%), whilst ADHD females emphasised relationship impact (24% vs. 12%) and emotional regulation (30% vs. 16%). Notably, ADHD females reported significantly higher self-understanding expectations than males (62% vs. 38%, p=0.016). Autism participants showed minimal gender differentiation, with both males and females equally focused on self-understanding (80% vs. 77%) and explaining to others (63% vs. 63%).

**Table 4:** Gender-stratified expectations by diagnostic group.

Expectation	ADHD male (n=50)	ADHD female (n=50)	Autism male (n=30)	Autism female (n=30)
Medication	46	48	3	0
Diagnostic certainty	44	28	10	10
Self-understanding	38	62*	80	77
Explaining to others	2	4	63	63
Cognitive function	44	28	47	43
Relationship impact	12	24	37	43
Emotional regulation	16	30	20	27

*p=0.016 for ADHD male versus female self-understanding comparison.

### Summary of key comparative findings

This comparative analysis reveals that adults seeking assessment for ADHD versus Autism articulate fundamentally distinct hopes. Five quantitative differences of meaningful magnitude emerged: (1) Medication expectations were 28 times higher in ADHD (47% vs. 1.7%); (2) Self-understanding expectations were three times higher in Autism (75% vs. 26%); (3) Explaining to others was 21 times more frequent in Autism (63% vs. 3%); (4) ADHD participants used deficit-focused self-descriptive language seven times more frequently; (5) The “racing mind” phenomenology appeared exclusively in ADHD responses.

The ADHD pathway is therefore characterised by a quest for legitimisation against stigma, expectation of pharmacological rescue from internal overwhelm, and an orientation toward resolving present crisis. The Autism pathway centres on identity development, psychosocial understanding, and explaining neurological difference to create mutual understanding. These divergent expectation patterns persist across demographic subgroups and suggest fundamentally different experiences of neurodivergence.

## Discussion

This comparative analysis of diagnostic expectations between adults referred for ADHD (n=100) and Autism (n=60) assessment reveals two fundamentally distinct orientations toward neurodevelopmental diagnosis. Adults presenting for an assessment of ADHD pursue a pathway of legitimisation and concrete intervention, seeking authoritative proof against stigma and immediate pharmacological solutions to acute crises. This is different from adults seeking an Autism assessment who pursue a pathway of identity development and social understanding, seeking frameworks for self-knowledge and tools for mutual comprehension within relationships and institutions. These contrasting orientations manifest across five conceptual dichotomies that reflect distinct cultural narratives, stigma experiences, and methods of seeking help with significant implications for clinical practice, service design, and theoretical understanding of neurodevelopmental diagnosis in adulthood.

### Five conceptual dichotomies in diagnostic expectations

The analysis reveals five fundamental dichotomies that distinguish ADHD from Autism expectations:

1. Intervention Paradigm: Pharmacological solution-seeking (ADHD) versus psychosocial understanding-seeking (Autism).
2. Epistemological Orientation: Diagnostic certainty as proof (ADHD) versus depth of self-understanding (Autism).
3. Social Positioning: Passive hope for understanding (ADHD) versus active education of others (Autism).
4. Self-Concept: Internalised deficit and moral failure (ADHD) versus neurological difference (Autism).
5. Phenomenological Experience: Internal cognitive overwhelm requiring quieting (ADHD) versus social-environmental mismatch requiring accommodation (Autism).

These dichotomies persist across demographic subgroups and suggest that ADHD and Autism are not merely different symptom clusters but represent fundamentally different ways of experiencing and framing neurodivergence.

### Medication expectations: the pharmacological intervention paradigm

The 28-fold difference in medication expectations between ADHD (47%) and Autism (1.7%) samples represents the most striking finding. Nearly half of ADHD participants spontaneously mentioned medication as a primary expectation, substantially exceeding rates in previous qualitative ADHD literature where medication typically emerges as one expectation among many (44). This prevalence reflects a deeply embedded narrative positioning ADHD as a pharmacologically treatable condition, reinforced by media coverage emphasising medication as first-line treatment, extensive direct-to-consumer marketing in some jurisdictions, and well-established evidence for stimulant efficacy (45).

The treatment mismatch theme, wherein 12% of ADHD participants explicitly mentioned current antidepressant treatment with inadequate efficacy, is important. Participants were clear even before diagnosed that they required ADHD-specific medication rather than antidepressants, with some naming specific medications. This pattern suggests many adults enter ADHD diagnostic pathways after unsuccessful treatment for depression or anxiety, viewing an ADHD diagnosis as necessary for accessing appropriate pharmacological intervention. This fits with research demonstrating that ADHD is usually accompanied by other psychiatric comorbidities for which the person may have been inappropriately treated instead of ADHD (46).

The virtual absence of medication expectations in the Autism sample (1.7%) reflects Autism’s current treatment landscape. Whilst off label medications are commonly prescribed for co-occurring conditions in autistic adults (47) no pharmacological treatment targets core Autism features. The neurodiversity movement’s influence may additionally shape expectations away from medical intervention and toward acceptance and adjustments (48).

These divergent medication expectations reflect fundamentally different intervention paradigms with ADHD positioned as a treatable medical condition with pharmacological solutions, Autism positioned as a difference requiring understanding and accommodation rather than treatment.

We also acknowledge that the magnitude of this finding must be interpreted with reference to the differential treatment landscape. Several pharmacological treatments are licensed for adult ADHD, whereas no agent is licensed for the core features of adult Autism, and a meaningful proportion of the expectation asymmetry is therefore a rational response to this structural reality. The cultural-narrative factors discussed above operate alongside, rather than in place of, this practical consideration.

In addition, we acknowledge that the clinical context in which the question was posed being a triage and screening appointment within a service explicitly designated for ADHD assessment may itself have foregrounded medication for ADHD-cohort participants in a way that an Autism-assessment context would not, simply because medication is part of the post-diagnostic conversation in one pathway and not in the other. This is a contextual contributor to the expectation asymmetry rather than an alternative explanation, and it operates alongside the cultural-narrative and treatment-landscape factors already discussed.

### Self-understanding versus diagnostic certainty: epistemological orientations

Autism participants showed threefold higher rates of self-understanding expectations (75%) compared to ADHD participants (26%). ADHD participants more frequently sought diagnostic certainty, a definitive “yes or no” answer (31% vs 10%) rather than a deep understanding of the condition.

This distinction suggests different epistemological relationships to diagnosis. Autism participants approached assessment as an opportunity for self-discovery and identity formation, seeking to understand “why I am the way I am.” The language reflects a depth orientation, participants wanted to understand Autism comprehensively, not merely confirm its presence. In contrast, ADHD participants demonstrated a certainty orientation as they wanted definitive confirmation that would legitimise difficulties and unlock access to treatment.

These orientations likely reflect differences in diagnostic timing, condition conceptualisation, and cultural narratives. Many Autism participants may have engaged in extended self-discovery processes before assessment, arriving with substantial self-knowledge seeking formal validation (49, 50). ADHD participants in this study more often described urgent crises requiring immediate solutions. The prominence of understanding in Autism expectations aligns with the neurodiversity movement discourse, which emphasises Autism as a different way of being rather than a disorder requiring treatment (51).

### Explaining to others: active educators versus passive recipients

The 21-fold difference in explaining to others (Autism: 63%; ADHD: 3%) is one of the study’s most theoretically significant findings. Nearly two-thirds of Autism participants anticipated using their diagnosis to explain Autism to family, partners, friends, colleagues, or broader communities, positioning themselves as active educators and self-advocates. Some explicitly mentioned educational goals, such as raising awareness within cultural communities with a limited understanding of Autism.

In contrast, only 3% of ADHD participants mentioned explaining to others, and when they did, it was framed passively, hoping others “might be more understanding” rather than actively planning to educate. Several factors may explain this pattern. First, Autism’s social communication features make explaining differences particularly salient. Second, the neurodiversity movement has created frameworks and language to explain Autism as a difference (35, 51). Third, Autism stigma may manifest more as social misunderstanding requiring active explanation, whilst ADHD stigma manifests more as moral judgment that explanation may not overcome (29, 52, 53).

Fourth, Autism diagnosis often occurs after years of social confusion and misattribution (19), creating an accumulated need to retrospectively explain oneself to long-puzzled family members. ADHD diagnosis may occur more acutely in response to specific crises, with less accumulated history requiring explanation.

### Deficit language versus difference language: the self-concept dichotomy

ADHD participants used deficit-focused, morally laden terminology such as “stupid,” “lazy,” “insane,” “crazy,” “broken,” “shit as a person” seven times more frequently than Autism participants. This striking difference reveals distinct manifestations of stigma. ADHD stigma appears to be internalised as moral failure and intellectual deficiency (29).

In contrast, Autism participants predominantly used identity-neutral, difference-focused language such as “the way I am,” “why I am the way I am,” “what makes me different.” This language difference likely reflects how conditions are culturally framed. ADHD symptoms inattention, impulsivity, disorganisation, map onto morally laden personality attributes in Western culture. A child who cannot sit still is “naughty,” an adult who misses deadlines is “lazy.” These moral framings become internalised over years of negative feedback.

Autism, whilst heavily stigmatised, is more often framed as “weirdness” or social oddity rather than moral failure. The neurodiversity movement has also provided alternative frameworks that position Autism as a neurological difference rather than a deficiency (35, 51). Although aligned, that movement has yet reframed ADHD stigma at scale, leaving people vulnerable to moral interpretations.

### Racing mind phenomenology: a distinctive ADHD experience

Ten per cent of ADHD participants spontaneously described this experience using vivid speed metaphors such as “1000mph,” “100mph,” “sped up” and explicit desires for mental quietening and peace. This theme was utterly absent from Autism responses (p=0.008), suggesting a distinctive phenomenological experience in ADHD.

Participants described relentless cognitive activity, inability to “switch off,” and overwhelming internal experience that medication might calm. Notably, this theme increased dramatically with age, reaching 71% in adults over 51, who shifted from achievement-focused to quality-of-life-focused expectations. Older adults wanted “peace for 10 minutes” rather than career success.

The absence of racing mind language in Autism is theoretically interesting. Whilst autistic people certainly experience cognitive and sensory overwhelm, they did not spontaneously describe this using speed metaphors or frame it as a primary problem requiring slowing or quietening. This may reflect different underlying mechanisms or different intervention expectations.

### Temporal orientations and institutional gatekeeping

Participants’ expectations followed a present-crisis to future-relief trajectory. They described current inability to cope, worsening situations, and urgent need for solutions. The temporal focus was narrow and immediate: solve the current crisis.

Autism participants demonstrated a past confusion to present understanding leading to future acceptance trajectory. They reflected on lifelong puzzlement about their differences, sought present understanding, and anticipated future self-acceptance. The temporal arc was broader and more developmental.

Ten per cent of ADHD participants explicitly mentioned institutional requirements for formal diagnosis before support provision, employers or universities stating they “will not give support unless a diagnosis.” This gatekeeping language appeared twice as frequently in ADHD as in Autism (10% vs 5%) and was framed more urgently.

### Gender and age patterns

Gender differences emerged more clearly within diagnostic groups than between them. ADHD males emphasised they wanted to know if they had the diagnosis and how it affects their cognitive function, whilst ADHD females emphasised relationship impact and emotional regulation. These patterns align with literature suggesting different ADHD presentations by gender (54). In the Autism sample, gender differences were minimal, with both males and females equally focused on self-understanding and explaining to others.

Age-stratified analysis revealed life stage-appropriate expectations. Younger adults emphasised educational support, middle-aged adults focused on workplace performance and parenting concerns, and older adults shifted toward life retrospective understanding and quality of life over achievement.

We do not extend the analysis of female ADHD presentations into the masking and camouflaging literature, because we do not accept that the construct, as developed in Autism research, applies to adult ADHD in the same form. The empirical and conceptual validity of camouflaging in ADHD has been examined and found wanting in our prior work (57), and importation of an Autism-derived construct into ADHD without independent empirical foundation risks obscuring rather than illuminating the gender pattern observed here. The framing of female ADHD presentations in terms of relationship impact and emotional regulation is offered descriptively, on the basis of the participants’ own accounts, and is not intended to invoke or imply a masking mechanism.

### Implications for service design: the case for separate diagnostic pathways

Our findings provide empirical evidence against combined neurodevelopmental pathways. The argument for separate pathways rests on five empirical foundations:

First, the 28-fold difference in medication expectations indicates that ADHD-seeking adults arrive anticipating pharmacological intervention, whilst Autism-seeking adults do not. A unified pathway providing generic information would either under-prepare ADHD participants for medication realities or inappropriately raise medication expectations in Autism participants.

Second, the 21-fold difference in explaining-to-others expectations demonstrates that Autism-seeking adults anticipate needing to educate family, friends, colleagues, and communities, whilst ADHD-seeking adults do not frame this as a priority. Post-diagnostic support for Autism requires explicit guidance on disclosure and communication strategies, which are primarily irrelevant to most ADHD participants.

Third, the language differences, ADHD participants’ deficit-focused, morally laden self-descriptions versus Autism participants’ identity-neutral difference language suggest fundamentally different forms of internalised stigma requiring different therapeutic approaches. ADHD pre-assessment counselling should explicitly address negative self-attributions and provide alternative explanatory frameworks.

Fourth, the racing mind phenomenology unique to ADHD constitutes a distinctive lived experience that requires specific clinical attention. This expectation is absent in Autism-seeking populations.

Fifth, the temporal orientation differences, ADHD’s crisis-relief trajectory versus Autism’s confusion-clarity-acceptance trajectory indicate different urgency levels and post-diagnostic timeframes requiring different service structures.

Beyond these specific expectation differences, separate pathways enable condition-specific expertise development among clinical staff. A generalist “neurodevelopmental” clinician attempting to maintain deep expertise across both domains would inevitably develop superficial knowledge in each.

The counter-argument for unified pathways typically emphasises three points: symptom overlap (55), high co-occurrence rates (56), and service efficiency (38). We do not address the scientific merits of the counter-argument here, but we note that, regarding symptom overlap, our data show that people self-select into ADHD versus Autism referral pathways based on their primary concerns and how they conceptualise their difficulties. We further note that some theoretical accounts (for example, those positing shared aetiological substrates or dimensional overlap between Autism and ADHD) have been advanced as a basis for combined pathways. We do not engage with these accounts in detail because the present study examines patient-articulated expectations rather than aetiology, and our argument for separate pathways rests on differences in what people seek from assessment rather than on claims about whether the two conditions share underlying mechanisms.

Regarding co-occurrence, the majority of referrals in our samples remained for single conditions. Services could maintain separate pathways whilst developing specialist provision for the minority presenting with explicit co-occurrence concerns. Regarding efficiency, combined pathways may appear administratively more straightforward but risk profound clinical inefficiency through mismatched provision.

The strongest argument for separate pathways is respect for how people conceptualise their own difficulties. ADHD-seeking adults frame their problems as internal dysregulation requiring treatment. Autism-seeking adults frame their experiences as different ways of being requiring understanding. These are not simply different symptoms but fundamentally different relationships to neurodevelopmental differences themselves.

Practically, separate pathways would involve distinct referral processes with condition-specific screening questions, supported by tailored pre-assessment information resources. They would rely on specialist clinical teams developing focused expertise, alongside differentiated post-diagnostic support groups, with clear mechanisms for additional assessment when co-occurrence becomes apparent.

### Clinical implications: operationalising condition-specific pathways

For ADHD pathways, pre-assessment materials should: (1) Set realistic medication expectations; (2) Address internalised stigma therapeutically; (3) Discuss institutional gatekeeping realities; (4) Normalise racing mind experiences; (5) Prepare for diagnostic uncertainty; (6) Address treatment mismatch concerns.

For Autism pathways, pre-assessment materials should: (1) Facilitate self-understanding processes; (2) Support communication planning; (3) Discuss neurodiversity perspectives; (4) Provide realistic timeframes for self-acceptance; (5) Clarify reasonable adjustments entitlements; (6) Acknowledge minimal medication expectations.

Post-diagnostic support should be similarly differentiated. ADHD support should emphasise medication titration, crisis intervention capacity, workplace accommodation negotiation, cognitive-behavioural approaches for internalised stigma, and executive function strategies. Autism support should emphasise identity integration, disclosure strategy development, sensory assessment, social relationships support, and neurodiversity-affirming therapeutic approaches.

## Limitations

Several limitations are acknowledged. First, samples came from a single NHS Trust, potentially limiting generalisability. Second, the Autism sample was smaller (n=60) than the ADHD sample (n=100). Third, responses were brief. Fourth, both samples included only people who successfully navigated referral pathways. Fifth, the cross-sectional design precludes understanding how expectations form over time. Sixth, we did not reanalyse the Autism data; instead, we compared our ADHD analysis with the published Autism findings.

Methodologically, using published Autism data as the comparator whilst analysing ADHD data *de novo* represents both a strength and a limitation. The strength is that the Autism analysis underwent rigorous peer review and publication. The limitation is that we could not ensure completely parallel analytical approaches, though both studies analysed the same Question 1 using Braun and Clarke’s reflexive thematic analysis.

Seventh, we acknowledge the theoretical possibility that asking participants about their expectations at the outset of an appointment could, in principle, influence the subsequent clinical session or participant responses thereafter. We are not aware of empirical evidence in the clinical or psychometric literature demonstrating that the asking about patient expectations at the beginning of an appointment biases the content of those expectations or the subsequent assessment. Moreover, any such effect would apply symmetrically across both cohorts, because participants in both samples were asked the same initial question in the same clinical context, and would therefore not plausibly account for the magnitude and direction of the comparative differences reported. Eliciting patients’ expectations is a standard and widely accepted element of competent clinical interviewing in adult psychiatry, and forms part of history-taking that informs formulation and the therapeutic alliance.

Eighth, although the two cohorts were asked the same first question using the same procedure within the same Trust, they were collected 12 to 18 months apart (Autism: January to June 2023; ADHD: December 2024 to February 2025) and differ in sample size (n=100 ADHD; n=60 Autism). No material service change occurred in the relevant assessment pathways during this period that would be expected to alter the content of participant expectations. The unequal sample sizes reflect the pragmatic reality that the Autism data were drawn from an already published study and the ADHD sample was assembled prospectively for the present comparison. In reflexive thematic analysis, the analytic aim is theoretical saturation rather than statistical power per se, and saturation was achieved in both samples. Where inferential statistics are reported, the tests used (chi-square with Bonferroni correction, or Fisher’s exact test for low cell counts) are appropriate for the given sample sizes, and the magnitude of the principal differences (for example, 47% versus 1.7% for medication; 75% versus 26% for self-understanding) is such that sample-size asymmetry is not a plausible explanation for the pattern of findings.

Ninth, because the expectations reported here were collected at the pre-assessment screening and triage stage, no confirmed diagnostic or formal comorbidity information was available at the point of data collection for either cohort. The study was not designed to quantify the effect of confirmed ADHD or Autism status, nor of confirmed co-occurrence, on expectations. The relevant classifying variable in the present work is therefore the referral pathway, which is the variable by which services are currently commissioned and organised. Future work comparing expectations among individuals with confirmed single-condition and confirmed dual-diagnosis outcomes would be a valuable complement to the present study.

## Future research directions

Future studies should look at whether people’s expectations influence how satisfied they feel after getting a diagnosis. Researchers should also test whether condition-specific pre-assessment counselling leads to better outcomes. It will be important to explore how expectations differ across cultures, and to understand where these expectations come from. Finally, services should be evaluated to see whether separate or combined assessment pathways lead to better satisfaction and outcomes for patients.

In addition, two further directions arise from the present analysis. First, future research should examine how previous exposure to, and identification with, neurodiversity-affirming frameworks shapes diagnostic expectations, particularly for Autism where such frameworks have developed more extensively than for ADHD. A design including measures of neurodiversity identification and community engagement would permit a more direct test of the cultural mechanisms hypothesised in this paper. Second, future studies should compare pre-assessment expectations with post-assessment diagnostic outcomes, including among individuals who receive confirmed single-condition diagnoses and those who receive confirmed dual diagnoses, so that the present findings in referral cohorts can be extended to diagnostically characterised populations.

## Conclusion

This study provides the first systematic empirical comparison of the diagnostic expectations of adults referred for ADHD and adults referred for Autism assessment, drawn from the same Trust and analysed against published Autism themes derived from an identical first question. The synthesis of the findings can be stated briefly. Adults entering the ADHD referral pathway arrive seeking legitimisation against moral attribution, pharmacological intervention for an experience of internal cognitive overwhelm, and a resolution of present crisis. Adults entering the Autism referral pathway arrive seeking self-understanding, an identity framework, and tools to explain difference to others. The two orientations are organised around five reproducible dichotomies, pharmacological versus psychosocial intervention paradigms, certainty versus depth, passive recipient versus active educator, deficit versus difference, internal overwhelm versus social-environmental mismatch that persist across age and gender strata.

The implication for service design follows from the synthesis rather than from any single finding. We suggest a combined neurodevelopmental pathway that takes the average of the two orientations will under-prepare ADHD participants for the realities of medication and stigma reframing, and will under-serve Autism participants on identity work, disclosure planning, and accommodation. Specifically, we recommend that commissioners and providers consider the following concrete steps: (i) retain or restore distinct ADHD and Autism referral streams, with condition-specific pre-assessment information; (ii) ringfence the share of any “neurodevelopmental” funding envelope that supports ADHD-specific pharmacological titration capacity, distinct from Autism-specific identity and communication support; (iii) develop separate post-diagnostic support offers, with explicit protocols for transfer between pathways where co-occurrence becomes apparent during assessment; and (iv) commission outcome monitoring that compares satisfaction and functional outcomes between separated and combined provision, so that the present argument can be tested empirically rather than settled administratively.

Theoretically, the findings indicate that ADHD and Autism are not interchangeable members of a single neurodevelopmental category at the level of patient meaning making, even where they may overlap at the level of symptoms or aetiology. The two conditions sit within distinct cultural framings, medical model and identity model that shape what people hope diagnosis will achieve. Services designed without reference to that distinction risk meeting nobody’s stated needs.

## Data Availability

The raw data supporting the conclusions of this article will be made available by the authors, without undue reservation.
